# Alcohol use disorder-related sick leave and mortality: a cohort study

**DOI:** 10.1186/1940-0640-8-3

**Published:** 2013-01-30

**Authors:** Felix Wedegaertner, Siegfried Geyer, Sonja Arnhold-Kerri, Nicola-Alexander Sittaro, Bert te Wildt

**Affiliations:** 1 Department of Psychiatry, Social Psychiatry and Psychotherapy, Hannover Medical School Centre for Mental Health, Carl-Neuberg-Str. 1, Hannover 30625, Germany; 2 Medical Sociology, Hannover Medical School, Carl-Neuberg-Str. 1, Hannover, 30625, Germany; 3 Hannover Life Re, Karl-Wiechert-Allee 50, Hannover, 30625, Germany

**Keywords:** Workers, Alcohol, Mortality, Gender, Addiction, Outpatients, Inpatients

## Abstract

**Background:**

Alcohol use disorders (AUDs) are associated with the highest all-cause mortality rates of all mental disorders. The majority of patients with AUDs never receive inpatient treatment for their AUD, and there is lack of data about their mortality risks despite their constituting the majority of those affected. Absenteeism from work (sick leave) due to an AUD likely signals worsening. In this study, we assessed whether AUD-related sick leave was associated with mortality in a cohort of workers in Germany.

**Methods:**

128,001 workers with health insurance were followed for a mean of 6.4 years. We examined the associations between 1) AUD-related sick leave managed on an outpatient basis and 2) AUD-related psychiatric inpatient treatment, and mortality using survival analysis, and Cox proportional hazard regression models (separately by sex) adjusted for age, education, and job code classification. We also stratified analyses by sick leave related to three groups of alcohol-related conditions (all determined by *International Classification of Diseases* 9^th^ ed. (ICD-9) codes): alcohol abuse and dependence; alcohol-induced mental disorder; and alcohol-induced medical conditions.

**Results:**

Outpatient-managed AUD-related sick leave was significantly associated with higher mortality (hazard ratio (HR) 2.90 (95% Confidence interval (CI) 2.24-3.75) for men, HR 5.83 (CI 2.90-11.75) for women). The magnitude of the association was similar for receipt of AUD-related psychiatric inpatient treatment (HR 3.2 (CI 2.76-3.78) for men, HR 6.5 (CI 4.41-9.47) for women). Compared to those without the conditions, higher mortality was observed consistently for outpatients and inpatients across the three groups of alcohol-related conditions. Those with alcohol-related medical conditions who had AUD-related psychiatric inpatient treatment appeared to have the highest mortality.

**Conclusions:**

Alcohol use disorder-related sick leave as documented in health insurance records is associated with higher mortality. Such sick leave does not necessarily lead to any specific AUD treatment. Therefore, AUD-related sick leave might be used as a trigger for insurers to intervene by offering AUD treatment to patients to try to reduce their risk of death.

## Background

Alcohol use disorders (AUDs) are prominent causes of morbidity and mortality, occurring in 4-9% of the population in any given year [1] and accounting for about 5% of all disability [2]. Alcohol-dependent patients have the highest standardized mortality ratio of all patients who receive inpatient psychiatric treatment [3,4]. Reliability of this estimate is low, because most individuals with AUDs receive no treatment for their disorder [5]. Past research suggests that a major reason for this is that individuals with AUDs do not perceive a need for treatment [6]. How can these undetected cases be brought to light? A strong indicator for the presence of an AUD is getting certified as unfit for work because of an alcohol-related diagnosis. Getting certified unfit for work is not necessarily followed by a specific treatment intervention, but shows impairment in social functioning typical for alcohol consumption patterns that get out of hand. As outpatient diagnoses are missing from most large epidemiological datasets, studies of the long-term outcome of these patients have been scarce.

German statutory health insurance clients represent 90% of the employed population and legislation ensures that any illness diagnosed and all periods of work incapacity are recorded and transmitted to the insurers. Workers who call in sick need to obtain a doctor’s certificate by the third day documenting the medical reason. This short interval between falling ill and a mandatory doctor’s visit makes the resulting insurer’s dataset suitable for studying outpatient diagnoses and to evaluate, if case properties derived from outpatient diagnoses are suited to trigger preventive interventions. The fact that clients are typically insured for long periods makes it possible to analyse the long-term outcome in terms of mortality. A diagnosis is only recorded when and if patients got certified as unfit for work and not if they are ill but never miss a day of work.

The *International Classification of Diseases,* 9^th^ ed. (ICD-9) used by insurers offers several diagnosis codes that are not purely descriptive, but ask the physician to make an assumption towards the cause of the illness. These diagnosis descriptions typically contain the phrase “induced by alcohol” (or similar attribution). While it is known that alcohol-dependent patients often have medical comorbidity and a high risk of dying from many diseases [4], it is of interest whether these mental or medical disorders induced by alcohol, which are only diagnosed if the physician specifically considers an alcohol etiology, further worsen the outcome.

We assessed whether AUD-related sick leave was associated with mortality in a cohort of workers in Germany. Finding an association would be the first step in determining whether such information might be used by insurers to decrease morbidity and mortality associated with AUDs among workers.

## Methods

### Data and subjects

Calculations were done on a dataset from the Mettmann Regional Office of the AOK Rheinland (a German public health insurance company), which contained the following details of insured individuals: data on claims for work incapacity, including diagnoses, duration of incapacity, identities, age, sex, highest attained education, job code classification and employment status (retired or employed) and, if applicable, date of death. Workers who were absent from work had to provide a medical certificate by the third day of absence. All medical reasons for work incapacity were passed on to the insurance company, which also keeps accurate data about hospital admissions and dates of death. The period of documentation extended from January 1, 1987 to October 31, 1996.

Insured individuals were only included in the study if they had been insured for a minimum period of 365 days and were 15 to 74 years old. Also, only subjects that were under obligation to present a medical certificate if they fell ill were included. This basically restricted the sample to workers and employees. Excluded cases consisted mainly of nonworking spouses, older retirees, and children.

### Independent variables

Subjects were considered to have an alcohol-related condition if they had any of the following:

I. Alcohol use disorders

Alcohol dependence syndrome (ICD-9 303.x)

Nondependent alcohol abuse (ICD-9 305.0)

II. Alcohol induced mental disorders (ICD-9 291.x)

III. Alcohol induced medical conditions:

Alcoholic gastritis (ICD-9 535.3)

Alcoholic fatty liver (ICD-9 571.0)

Acute alcoholic hepatitis (ICD-9 571.1)

Alcoholic cirrhosis of liver (ICD-9 571.2)

Alcoholic liver damage unspecified (ICD-9 571.3)

Alcoholic polyneuropathy (ICD-9 357.5)

Alcoholic cardiomyopathy (ICD-9 425.5)

The main independent variables of interest were 1) AUD-related sick leave managed on an outpatient basis and 2) AUD-related psychiatric inpatient treatment. The insured individuals were counted as a case of “outpatient treatment” if at least one period of absence from work resulting from the above alcohol-related conditions was documented in the observation period, but no inpatient psychiatric stay. A case of inpatient treatment was defined by at least one inpatient psychiatric stay with one of the above alcohol-related conditions documented (regardless of whether it was preceded or followed by sick-leave managed as an outpatient). Inpatient psychiatric treatments included treatments in specialist mixed neurological-psychiatric departments and those that were started in other medical clinics and led to the patient being transferred to inpatient psychiatric treatment.

### Dependent variable

The dependent variable was death from any cause.

### Analyses

Statistical analysis was performed with IBM SPSS® statistical software, version 18. On a descriptive level, survival (mortality) was calculated and displayed using Kaplan- Meier curves. Each insured individual was included in the study with their own observation period in days, the duration of their insurance. This insurance time period ended either with the death of the subject or because the time of observation ended. Employing Cox proportional hazards regression models, the mortality risk of persons with “inpatient” or “outpatient” status (AUD-related sick leave managed on an outpatient basis and AUD-related psychiatric inpatient treatment) was compared with the risk of the controls (those with no [outpatient or inpatient] AUD-related sick leave). Previous studies have shown a positive relationship between alcoholism and employment for women [7,8]. Therefore, the models were calculated separately for men and women and controlled for age, education and job code classification. Interactions between sex and the independent variables of interest were calculated if variables of interest showed significant differences in main effects models. We also stratified analyses by sick leave related to the three groups of alcohol-related conditions: alcohol abuse and dependence; alcohol-induced mental disorder; and alcohol-induced medical conditions. Legal basis of data transmission and analysis was section 287 of the German Social Code Book V. No individuals were examined. Therefore, ethics committee approval was not necessary.

## Results

### Characteristics of the sample

In total, the insurance records of 417,496 insured individuals were available. Of those, 4.837 had missing data, 102,102 had not been insured a minimum of 365 days, 98.637 were under 15 or over 74 years of age, and 83.919 were clients who were not under any obligation to present a medical certificate if they fell ill. The remaining 128,001 records were used for the analysis of mortality (Table 1). The mean observation period was 6.4 years. The age and sex distribution as well as the proportion of employees in the sample were consistent with the distribution of employees with statutory health insurance in Germany during the period of observation [9].

**Table 1:** Descriptive statistics of the sample

**Variable**	**Value**		
Observed cases	128,001 (all employed persons)		
Mean observation period	2350 days (6.4 years)		
Sex	male	85,502	66.8%
	female	42,499	33.2%
Age	Point of observation during study period	Start	End
	Mean	34.99	41.67
	Standard deviation	12.36	13.58
	Minimum	15	16
	Maximum	64	74
Education	General school certificate		
	(9 years of schooling)	40,498	31.6%
	Intermediate secondary school certificate		
	(10 years of schooling)	52,208	40.8%
	Qualifying high school degree		
	(13 years of schooling)	2,786	2.2%
	University degree	1,090	0.9%
	Not known	29,271	22.9%
Job code classification	Occupations with little or no qualification	57,097	57.9%
	Qualified manual occupations, skilled workers	28,778	29.1%
	Qualified white-collar occupations	11,700	11.9%
	Middle-management and management positions	1,155	1.2%
	Not known	29,271	22.9%

### AUDs and mortality

The numbers of subjects, years of observation, and number and proportion who died appear in Table 2.

**Table 2:** Mortality of workers stratified by sex, alcohol-related diagnoses and AUD-related sick leave treatment received

**Early mortality**						
Sex	Alcohol-related condition	Type of treatment*	Years of observation	Number of subjects	Number of deaths	Deaths per 1000 years of observation
Men	Alcohol use disorder	Outpatient treatment	5106	670	59	11.6
		Inpatient treatment	12663	1594	166	13.1
	Controls	None	551130	83238	2466	4.5
	Alcohol induced mental disorders	Outpatient treatment	5637	694	85	15.1
		Inpatient treatment	2068	260	87	42.1
	Controls	None	561194	84548	2519	4.5
	Alcohol induced medical conditions	Outpatient treatment	1324	168	25	18.9
		Inpatient treatment	1643	216	35	21.3
	Controls	None	565932	85118	2631	4.6
Women	Alcohol use disorder	Outpatient treatment	962	127	8	8.3
		Inpatient treatment	2061	268	28	13.6
	Controls	None	283141	42104	557	2.0
	Alcohol-related mental disorders	Outpatient treatment	1331	156	11	8.3
		Inpatient treatment	515	64	16	31.1
	Controls	None	284318	42279	566	2.0
	Alcohol induced medical conditions	Outpatient treatment	181	22	3	16.6
		Inpatient treatment	321	40	3	9.3
	Controls	None	285662	42437	587	2.1
Both	Alcohol use disorder	Outpatient treatment	6068	797	67	11.0
		Inpatient treatment	14724	1862	194	13.2
	Controls	None	834271	125342	3023	3.6
	Alcohol-related mental disorders	Outpatient treatment	6968	850	96	13.8
		Inpatient treatment	2583	324	103	39.9
	Controls	None	845512	126827	3085	3.6
	Alcohol induced medical conditions	Outpatient treatment	1505	190	28	18.6
		Inpatient treatment	1964	256	38	19.3
	Controls	None	851594	127555	3281	3.9

*Outpatient treatment = Alcohol-use-disorder (AUD)-related sick leave managed on an outpatient basis. Inpatient = AUD-related psychiatric inpatient treatment. None=Neither outpatient or inpatient treatment.

**Figure 1:**
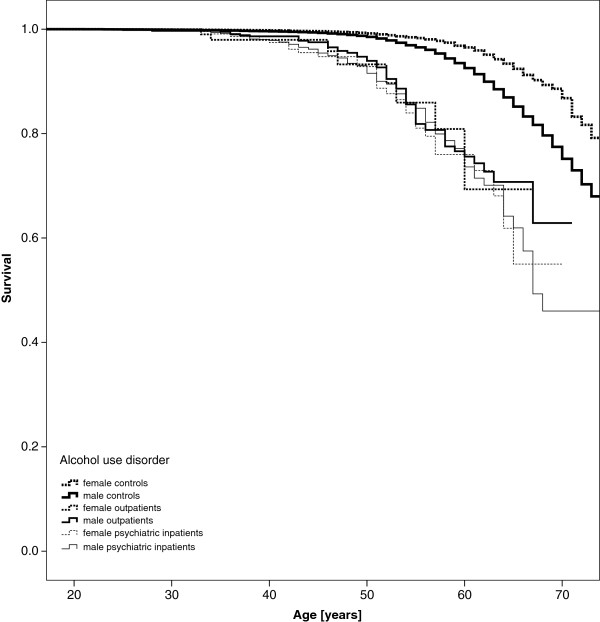
All-cause mortality - Kaplan-Meier curves of patients with alcohol use disorder vs. controls.

**Figure 2:**
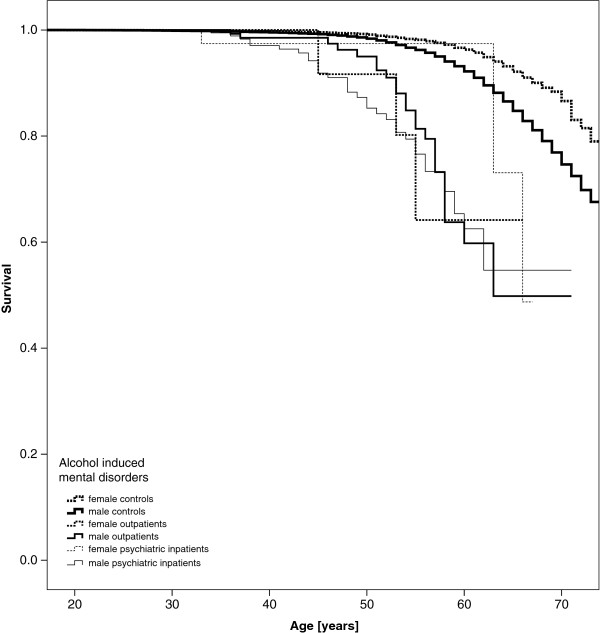
All-cause mortality - Kaplan-Meier curves of patients with alcohol induced mental disorders vs. controls.

**Figure 3:**
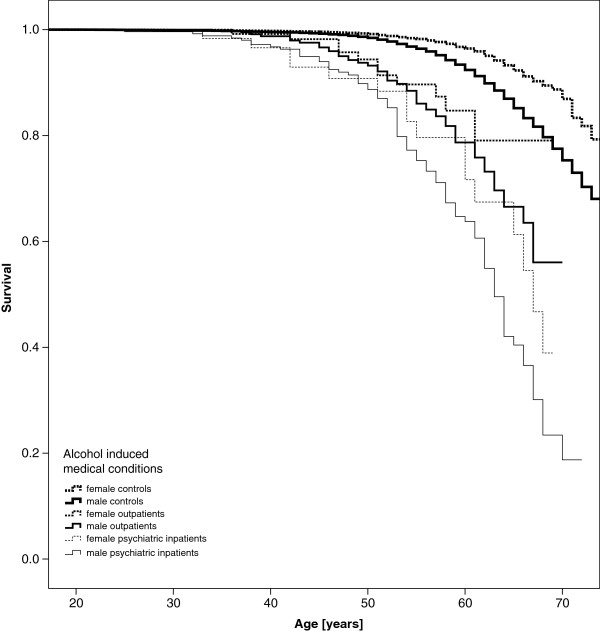
All-cause mortality - Kaplan-Meier curves of patients with alcohol induced medical conditions vs. controls.

Kaplan Meier curves appear in Figure 1 for those with AUD-related psychiatric inpatient treatment, AUD-related sick leave managed on an outpatient basis and for controls who experienced neither. Curves are stratified by sex. Women appear to have better survival than men, and men and women with either inpatient or outpatient treatment have worse (though similar to each other) survival than those with no AUD-related sick leave. Results were similar for curves displaying survival of those with and without sick leave and inpatient treatment related to alcohol-induced mental disorders and medical conditions Figures 1, 2 and 3.

Unsurprisingly, patients in almost all strata who had alcohol-related conditions and sick leave had higher mortality (see Table 3). Alcohol use disorders were the most frequent of all alcohol-related diagnoses (Table 2). Men and women who received inpatient psychiatric treatment for alcohol-induced medical conditions had the highest relative hazard of mortality, although confidence intervals overlapped with those for alcohol-induced mental disorders. In general, women with alcohol-related sick leave had a greater relative hazard of death than men, but this sex-interaction was significant only for receipt of AUD-related psychiatric inpatient treatment.

**Table 3:** Mortality of alcohol use disorder, alcohol induced mental disorder and alcohol induced medical condition-related sick leave managed as an outpatient or by psychiatric inpatient treatment

**Gender**	**Illness**	Type of treatment **	Hazard ratio*	**Confidence interval**
Men	Alcohol addiction and misuse	Outpatient only	2.90	2.24–3.75
		Inpatient	3.23 ^†††^	2.76–3.78
	Alcohol induced mental disorders	Outpatient only	4.14	2.79–6.13
		Inpatient	4.52	3.24–6.31
	Alcohol induced medical conditions	Outpatient only	2.96	2.38–3.67
		Inpatient	6.35	5.13–7.87
Women	Alcohol addiction and misuse	Outpatient only	5.83	2.90–11.75
		Inpatient	6.46 ^†††^	4.41–9.47
	Alcohol induced mental disorders	Outpatient only	8.75	2.81–27.22
		Inpatient	3.10	1.00–9.66
	Alcohol induced medical conditions	Outpatient only	4.18	2.30–7.60
		Inpatient	8.82	5.36–14.53
Both	Alcohol addiction and misuse	Outpatient only	3.46	2.71–4.41
		Inpatient	3.85 ^†††^	3.33–4.45
	Alcohol induced mental disorders	Outpatient only	4.88	3.37–7.09
		Inpatient	4.74	3.45–6.54
	Alcohol induced medical conditions	Outpatient only	3.37	2.75–4.13
		Inpatient	7.04	5.79–8.58

* Hazard ratios adjusted for age, education, job code classification.

**Outpatient treatment = Alcohol-use-disorder (AUD)-related sick leave managed on an outpatient basis. Inpatient = AUD-related psychiatric inpatient treatment. None=Neither outpatient or inpatient treatment.

^†††^Denotes a statistically significant gender interaction (HR 2.16, CI 1.43-3.25).

## Discussion

The main findings are that workers with alcohol-related sick leave and either outpatient or inpatient psychiatric management have higher mortality than workers who do not. Additional analyses suggest that the relative hazard is greater for women, and for those who receive inpatient treatment associated with alcohol-related medical conditions. Hazard ratios for alcohol-induced mental disorders were also often higher than those for AUDs alone though confidence intervals overlapped.

Among those with AUD-related sick leave, whether inpatient psychiatric care was applied or not did not seem to have an influence on mortality. A clear protective effect of inpatient treatment on mortality could not be seen, but neither could a significantly higher mortality in inpatients. This is slightly counter-intuitive, as one would have expected a higher mortality in psychiatric inpatients, following the train of thought that these patients should be more strongly affected than those who are in outpatient treatment. On the other hand, a protective effect might also have been expected if inpatient treatment for alcohol addiction was more effective in causing prolonged abstinence.

Alcohol consumption has a strong effect on work performance and is thus strongly associated with job loss [10]. It has also been proven that job loss is associated with increased frequency of alcohol addiction [11]. When combining this information with the fact that employment in itself is a deterrent for seeking inpatient treatment [12], it is not surprising that the employed are a minority, about 20%, among alcohol addicted inpatients [13].

Many studies show a U- or J-shaped relationship between alcohol intake and mortality [14]. High alcohol intake is strongly associated with both elevated mortality [14,15] and absence from work [16-18]. Sickness absence from work is a predictor of mortality [19]. To our knowledge, mortality rates of those who have periods of sick leave due to AUDs without seeking inpatient treatment have not been published, although those patients are the vast majority of those affected by AUDs [20]. Most authors who compare diagnosis-specific sickness absence from work do not differentiate between the psychiatric diagnoses or whether patients receive additional inpatient treatment or not [21-23]. It has previously been suggested that absence from work due to a psychiatric disorder may help to identify individuals at risk of premature mortality and serve to monitor workers’ health [23]. Results of this study support this finding.

Published results for excess mortality of patients who receive inpatient treatment for AUDs in a similar study setting [4] were comparable to those in this study. Mortality rates published by other authors were lower when a shorter follow-up period was used [24] and when the diagnosis was made independently of a form of treatment [25].

Alcohol use disorders are among those psychiatric disorders with the highest mortality and impact on public health [4,24-27]. For inpatient men, a standardized mortality ratio (SMR) of 3.64 could be shown, while female patients had an SMR of 3.58 for mortality due to natural causes [4]. It can be replicated that both genders have exceptionally high excess mortality due to homicide, suicide, and accidents [3,28,29], up to an almost 17-fold increase. Notable gender differences are in the area of accidents [29] and suicides [27], for which women appear to have a higher risk. This study replicated the SMR for men, but we found a higher mortality for women than did previous authors. This may partly be due to the sampling employed in this study. By studying only workers and employees, the sample does not represent all women. Employed women have a more healthy lifestyle than unemployed women [30]. Better health of those who work can also be assumed. Nevertheless, given traditional western role models, employed women may find themselves under more stress than men when they have greater household responsibilities as well [31]. As men outnumbered women in the sample by 2:1, it is likely that selection mechanisms added to the possibly higher relative mortality among alcohol-addicted women in the sample.

### Strengths and weaknesses

Main strengths of this study are the large sample size, longitudinal data, and the fact that the study design allowed to calculate mortality outcomes for workers. A main weakness is under-diagnosis, which likely means that the mortality is underestimated and that the study is biased towards the null hypothesis. Among inpatients, a more severe addiction could have been assumed if they had alcohol-induced medical or mental conditions. Therefore, higher mortality in this substratum was not surprising. Still, the heterogeneity of this group limits the usefulness of “alcohol-related mental disorders” and “alcohol-related medical conditions” as surrogate parameters for severity and need for life-saving interventions.

Effects of psychiatric illness on mortality need to be discussed with caution, especially when they rely on outpatient diagnoses. Special consideration must be given to the circumstances under which the diagnoses were made for the purpose of this study. Strong under-reporting has to be taken into account in the outpatient setting. One has to expect that a doctor would have regarded an employee unfit for work because of an AUD only if the addiction was unmistakeable. Alcohol use disorders are associated with embarrassment, especially if it causes reduced performance in the workplace. Therefore, doctors may have tended to make a less stigmatizing diagnosis if at all possible. Although this limits the usefulness of sick leave data, it makes the parameter not entirely unsuitable. Missing a day of work is a clearly defined index event. Also, one has to ask what other parameter would indicate an alcohol problem getting out of hand other than a doctor’s diagnosis. Reduced social functioning is a factor of harmful use or addiction both in revision 9 and 10 of the *International Classifications of Diseases*, and that includes missed days at work.

Apart from this, the nature of the alcohol problems of workers and employees may be somewhat different from those of unemployed people. While it may be assumed that the unemployed have a greater risk of developing an alcohol problem that gets out of hand, it is unclear how to reach such people if they have no necessity to contact a doctor until complications from alcohol consumption are grave. In addition, the descriptive statistics of the sample suggests that blue-collar workers may have been over-represented in the sample. This limits the application of our results to employees as a whole.

Most published studies did not discern between *psychiatric* inpatient stays and inpatient stays as a whole. In particular, the large epidemiological Scandinavian studies rely solely on register data, which only reflects inpatient stays [3,4,27]. As a result, previously published mortality estimates have to be interpreted with the limitation that it remains unknown whether patients had a specific therapeutic intervention for their psychiatric diagnosis. Additionally, those who were solely outpatients are mostly not included in those studies. It is understandable from the perspective that this data simply does not exist in most epidemiological datasets, but the approach has its limitations, as it is known that the majority of alcohol-addicted patients never get admitted to an inpatient facility [32,33]. Even in our study, we saw that a large number of patients diagnosed with alcohol addiction and misuse or an alcohol-induced medical or mental condition never received treatment in a psychiatric hospital. In addition, there is strong social inequality in the utilization of in- and outpatient treatment for addiction, with those subjects of higher social status avoiding psychiatric inpatient hospitalization [34]. The selection mechanisms at work for inpatient psychiatric treatment may explain why the authors could not find a relevant difference in mortality when outpatient and inpatient groups were compared.

However, the biggest problem with the dataset is that the majority of alcohol-addicted patients are not diagnosed as such [35]. Therefore, the impact of alcohol addiction on a society as a whole cannot be derived from these results. Employee assistance programs (EAPs) may be more sensitive and more effective, but routine data analysis for the index events defined in this study has the great advantage, in that all clients can be screened regardless of whether EAP programs exist at a given workplace. Confidentiality rules out that insurers pass their knowledge of index events to EAPs, but it is self-evident that the best results may be achieved by employers, health insurers, and therapeutic personnel working together when addiction surfaces in a work context.

## Conclusions

These results indicate that missed days at work because of AUDs is associated with mortality. The implications of these results are that health insurance data on alcohol-related conditions associated with sick leave might be useful for identifying people who might not otherwise come to clinical attention. General practitioners should consider whether more intensive multidisciplinary treatment is warranted if a patient presents with the request to be certified as unfit for work and the doctor diagnoses an alcohol-related problem as causative.

## Competing interests

The authors state they have no competing interests.

## Authors’ contribution

FW wrote the manuscript, did statistical analysis. SG corrected the manuscript, provided the data set. SAK processed the data set for analysis. NAS provided support in the interpretation of the results. BtW did the initial conception of the study. All authors read and approved the final manuscript.

## References

[B1] Kessler RC Wai TC Demler O Walters EE Prevalence, severity, and comorbidity of 12-month DSM-IV disorders in the national comorbidity survey replication Arch Gen Psychiatry 2005 62 617 627 10.1001/archpsyc.62.6.617 15939839 PMC2847357

[B2] Murray CJL Lopez AD Evidence-based health policy - lessons from the global burden of disease study Science 1996 274 740 743 10.1126/science.274.5288.740 8966556

[B3] Hiroeh U Appleby L Mortensen PB Dunn G Death by homicide, suicide, and other unnatural causes in people with mental illness: a population-based study Lancet 2001 358 2110 2112 10.1016/S0140-6736(01)07216-6 11784624

[B4] Hiroeh U Kapur N Webb R Dunn G Mortensen PB Appleby L Deaths from natural causes in people with mental illness: a cohort study J Psychosom Res 2008 64 275 283 10.1016/j.jpsychores.2007.09.008 18291242

[B5] Edlund MJ Booth BM Feldman ZL Perceived need for treatment for alcohol use disorders: results from two national surveys Psychiatr Serv 2009 60 1618 1628 10.1176/appi.ps.60.12.1618 19952152 PMC2859201

[B6] Grant BF Barriers to alcoholism treatment: reasons for not seeking treatment in a general population sample Journal of Studies on Alcohol 1997 58 365 371 9203117 10.15288/jsa.1997.58.365

[B7] Mullahy J Sindelar JL Women and work: tipplers and teetotalers Heal Econ 1997 6 533 537 10.1002/(SICI)1099-1050(199709)6:5<533::AID-HEC296>3.0.CO;2-F 9353657

[B8] Wilsnack SC Klassen AD Schur BE Wilsnack RW Predicting onset and chronicity of women’s problem drinking: a five-year longitudinal analysis Am J Public Health 1991 81 305 318 10.2105/AJPH.81.3.305 1994739 PMC1405008

[B9] Federal Statistic Office Erwerbstätige - wirtschaftliche und berufliche gliederung, berufsausbildung economic and professional organization training 2003 Wiesbaden, Bonn, Berlin: Selbstverlag

[B10] Henkel D Unemployment and substance use: a review of the literature (1990–2010) Curr Drug Abuse Rev 2011 4 4 27 10.2174/1874473711104010004 21466502

[B11] Jin RL Shab CP Svoboda TJ The impact of unemployment on health: a review of the evidence CMAJ 1995 153 529 540 7641151 PMC1487417

[B12] Hajema KJ Knibbe RA Drop MJ Social resources and alcohol-related losses as predictors of help seeking among male problem drinkers J Stud Alcohol Drugs 1999 60 120 129 10.15288/jsa.1999.60.120 10096317

[B13] Freyer-Adam J Gaertner B Rumpf HJ John U Hapke U Alcohol-dependent inpatients who receive general hospital care vs. Detoxification in psychiatric care and alcohol problem 1 year later Addict Behav 2010 35 756 763 10.1016/j.addbeh.2010.03.002 20395058

[B14] Poikolainen K Alcohol and mortality: a review J Clin Epidemiol 1995 48 455 465 10.1016/0895-4356(94)00174-O 7722599

[B15] Mattisson C Bogren M Ojehagen A Nordstrom G Horstmann V Mortality in alcohol use disorder in the lundby community cohort-a 50 year follow-up Drug Alcohol Depend 2011 118 141 147 10.1016/j.drugalcdep.2011.03.008 21474255

[B16] Vahtera J Poikolainen K Kivimaki M Ala-Mursula L Pentti J Alcohol intake and sickness absence: a curvilinear relation Am J Epidemiol 2002 156 969 976 10.1093/aje/kwf138 12419770

[B17] Upmark M Moller J Romelsjo A Longitudinal, population-based study of self reported alcohol habits, high levels of sickness absence, and disability pensions J Epidemiol Community Health 1999 53 223 229 10.1136/jech.53.4.223 10396548 PMC1756858

[B18] Marmot MG North F Feeney A Head J Alcohol consumption and sickness absence: from the whitehall II study Addiction 1993 88 369 382 10.1111/j.1360-0443.1993.tb00824.x 8461854

[B19] Vahtera J Pentti J Kivimaki M Sickness absence as a predictor of mortality among male and female employees J Epidemiol Community Health 2004 58 321 326 10.1136/jech.2003.011817 15026447 PMC1732735

[B20] Kohnke M Treatment of alcoholism in Germany Ann Acad Med Stetin 2009 55 97 99 discussion 99 20349599

[B21] Ferrie JE Vahtera J Kivimaki M Westerlund H Melchior M Alexanderson K Head J Chevalier A Leclerc A Zins M Diagnosis-specific sickness absence and all-cause mortality in the GAZEL study J Epidemiol Community Health 2009 63 50 55 19039005 10.1136/jech.2008.074369 PMC2695575

[B22] Head J Ferrie JE Alexanderson K Westerlund H Vahtera J Kivimaki M Diagnosis-specific sickness absence as a predictor of mortality: the whitehall II prospective cohort study BMJ 2008 337 855 858 10.1136/bmj.a1469 PMC2563263 18832415

[B23] Melchior M Ferrie JE Alexanderson K Goldberg M Kivimaki M Singh-Manoux A Vahtera J Westerlund H Zins M Head J Does sickness absence due to psychiatric disorder predict cause-specific mortality? a 16-year follow-up of the GAZEL occupational cohort study Am J Epidemiol 2010 172 700 707 10.1093/aje/kwq186 20732935 PMC2938268

[B24] Dawson DA Alcohol consumption, alcohol dependence, and all-cause mortality Alcohol Clin Exp Res 2000 24 72 81 10.1111/j.1530-0277.2000.tb04556.x 10656196

[B25] Chwastiak LA Rosenheck RA Desai R Kazis LE Association of psychiatric illness and all-cause mortality in the national department of veterans affairs health care system Psychosom Med 2010 72 817 822 10.1097/PSY.0b013e3181eb33e9 20639387 PMC2950891

[B26] Balakrishnan R Allender S Scarborough P Webster P Rayner M The burden of alcohol-related ill health in the united kingdom J Pub Health 2009 31 366 373 10.1093/pubmed/fdp051 19493915

[B27] Hannerz H Borga P Borritz M Life expectancies for individuals with psychiatric diagnoses Pub Health 2001 115 328 337 10.1016/S0033-3506(01)00471-1 11593442

[B28] Klatsky AL Armstrong MA Alcohol use, other traits, and risk of unnatural death: a prospective study Alcohol Clin Exp Res 1993 17 1156 1162 10.1111/j.1530-0277.1993.tb05221.x 8116824

[B29] McCaul KA Almeida OP Hankey GJ Jamrozik K Byles JE Flicker L Alcohol use and mortality in older men and women Addiction 2010 105 1391 1400 10.1111/j.1360-0443.2010.02972.x 20528808

[B30] Virtanen P Vahtera J Broms U Sillanmaki L Kivimaki M Koskenvuo M Employment trajectory as determinant of change in health-related lifestyle: the prospective HeSSup study Eur J Public Health 2008 18 504 508 10.1093/eurpub/ckn037 18515862

[B31] Thurston RC Sherwood A Matthews KA Blumenthal JA Household responsibilities, income, and ambulatory blood pressure among working men and women Psychosom Med 2011 73 200 205 10.1097/PSY.0b013e3182080e1a 21217097 PMC3038680

[B32] Aldama E Arino J Baliesteros J Segovia M Gutierrez M Use of alcoholism psychiatric services by male alcoholics in an 18-month follow-up Med Clinica 1996 106 768 775 8801395

[B33] Kohn R Saxena S Levav I Saraceno B The treatment gap in mental health care Bull World Health Organ 2004 82 858 866 15640922 PMC2623050

[B34] Geyer S Haltenhof H Peter R Social inequality in the utilization of in- and outpatient treatment of non-psychotic/non-organic disorders: a study with health insurance data Soc Psychiatry Psychiatr Epidemiol 2001 36 373 380 10.1007/s001270170027 11766967

[B35] Buchsbaum DG Buchanan RG Poses RM Schnoll SH Lawton MJ Physician detection of drinking problems in patients attending a general medicine practice J Gen Internal Med 1992 7 517 521 10.1007/BF02599456 1403208

